# Machine learning for screening of at-risk, mild and moderate COPD patients at risk of FEV_1_ decline: results from COPDGene and SPIROMICS

**DOI:** 10.3389/fphys.2023.1144192

**Published:** 2023-04-21

**Authors:** Jennifer M. Wang, Wassim W. Labaki, Susan Murray, Fernando J. Martinez, Jeffrey L. Curtis, Eric A. Hoffman, Sundaresh Ram, Alexander J. Bell, Craig J. Galban, MeiLan K. Han, Charles Hatt

**Affiliations:** ^1^ Division of Pulmonary and Critical Care Medicine, University of Michigan, Ann Arbor, MI, United States; ^2^ Department of Biostatistics, School of Public Health, University of Michigan, Ann Arbor, MI, United States; ^3^ Weill Cornell Medical College, New York, NY, United States; ^4^ Medical Service, VA Ann Arbor Healthcare System, Ann Arbor, MI, United States; ^5^ Department of Radiology, University of Iowa, Iowa City, IA, United States; ^6^ Department of Radiology, University of Michigan, Ann Arbor, MI, United States; ^7^ Department of Biomedical Engineering, University of Michigan, Ann Arbor, MI, United States; ^8^ Imbio Inc., Minneapolis, MN, United States

**Keywords:** chronic obstructive pulmonary disease, machine learning, computed tomography, lung function decline, quantitative imaging

## Abstract

**Purpose:** The purpose of this study was to train and validate machine learning models for predicting rapid decline of forced expiratory volume in 1 s (FEV_1_) in individuals with a smoking history at-risk-for chronic obstructive pulmonary disease (COPD), Global Initiative for Chronic Obstructive Lung Disease (GOLD 0), or with mild-to-moderate (GOLD 1–2) COPD. We trained multiple models to predict rapid FEV_1_ decline using demographic, clinical and radiologic biomarker data. Training and internal validation data were obtained from the COPDGene study and prediction models were validated against the SPIROMICS cohort.

**Methods:** We used GOLD 0–2 participants (*n* = 3,821) from COPDGene (60.0 ± 8.8 years, 49.9% male) for variable selection and model training. Accelerated lung function decline was defined as a mean drop in FEV_1_% predicted of > 1.5%/year at 5-year follow-up. We built logistic regression models predicting accelerated decline based on 22 chest CT imaging biomarker, pulmonary function, symptom, and demographic features. Models were validated using *n* = 885 SPIROMICS subjects (63.6 ± 8.6 years, 47.8% male).

**Results:** The most important variables for predicting FEV_1_ decline in GOLD 0 participants were bronchodilator responsiveness (BDR), post bronchodilator FEV_1_% predicted (FEV_1_.pp.post), and CT-derived expiratory lung volume; among GOLD 1 and 2 subjects, they were BDR, age, and PRM_lower lobes fSAD_. In the validation cohort, GOLD 0 and GOLD 1–2 full variable models had significant predictive performance with AUCs of 0.620 ± 0.081 (*p* = 0.041) and 0.640 ± 0.059 (*p* < 0.001). Subjects with higher model-derived risk scores had significantly greater odds of FEV_1_ decline than those with lower scores.

**Conclusion:** Predicting FEV_1_ decline in at-risk patients remains challenging but a combination of clinical, physiologic and imaging variables provided the best performance across two COPD cohorts.

## Introduction

Chronic obstructive pulmonary disease (COPD) is characterized by a variety of clinical phenotypes and disease courses that are often difficult to predict (Miravitlles et al., 2013; Mathioudakis et al., 2020). There is a pressing need to create actionable tools to assist clinicians and researchers in identifying patients who are at higher risk for accelerated lung function decline so that early, directed therapies can be appropriately initiated. In addition to conventional markers of lung function decline using pulmonary function testing (PFT) metrics, novel advanced chest imaging analytic techniques are currently being explored to identify high risk patients. Among these techniques are Parametric Response Mapping (PRM), which co-registers inspiratory and expiratory images to distinguish between normal lung, emphysema, and non-emphysematous air trapping (functional small airways disease, fSAD). Prior studies using PRM have demonstrated the association between fSAD and 5-year forced expiratory volume in 1 s (FEV_1_) decline, progression of emphysema and exacerbation risk (Bhatt et al., 2016; Han et al., 2017; Labaki et al., 2019).

Vascular remodeling is also prevalent among COPD patients and believed to be part of the pathogenesis of this disease. CT scans have been used to visualize changes in distal pruning of blood vessels (Rahaghi et al., 2019). The ratio of blood volume in vessels with a cross-sectional area < 5 mm^2^ to total blood vessel volume (TBV) has been proposed as an imaging biomarker and, consistent with this theory, has been shown to decrease as COPD progresses (Estepar et al., 2013). Increased airway wall thickness (AWT) has also been associated with more frequent COPD exacerbations (Han et al., 2011) and with greater FEV_1_ decline and development of airflow limitation in smokers (Mohamed et al., 2015). AWT is often measured using Pi10, the square root of an airway wall area with a 10 mm lumen perimeter (Nakano et al., 2005).

Predictive models have been widely used for prediction of clinically meaningful outcomes in subjects with COPD. These models have been used to identify factors that place patients at-risk-for exacerbations and hospital admissions and readmissions and to demonstrate the effect of smoking reduction on FEV_1_ decline (Simmons et al., 2005; Bahadori et al., 2009; Bertens et al., 2013). Predictive models have also been combined with deep learning methods to assist in staging COPD severity and predict disease progression using automated CT staging to quantify the degree of emphysema and air trapping visualized on images (Hasenstab et al., 2021). Predicting risk of mortality, both in patients admitted to the ICU with exacerbations (Jain et al., 2018) and those in the outpatient primary care setting using a variety of predictive modeling methods (Kiddle et al., 2020) has been extensively studied. However, prediction of FEV_1_ decline has remained challenging with currently available risk models.

The purpose of this study was to train and evaluate logistic regression prediction models for rapid FEV_1_ decline over a 5-year time span in ever-smoking participants of the Genetic Epidemiology of Chronic Obstructive Pulmonary Disease (COPDGene) study who were either at-risk-for COPD (smoking history but normal spirometry, GOLD 0) or with mild-moderate COPD (GOLD 1–2). We used a variable importance methodology to select and rank a subset of variables that were important for prediction of rapid FEV_1_ decline (defined as a drop in FEV_1_% predicted of > 1.5%/year). Models using only imaging biomarkers and data readily available from the Digital Imaging and Communications in Medicine (DICOM) header (i.e., age, sex) were also trained and compared to full data models to determine if data captured only in a CT scan has adequate predictive value. Finally, we externally validated these prediction models in the large Subpopulations and Intermediate Outcome Measures in COPD (SPIROMICS) cohort.

## Materials and methods

This study is a retrospective analysis of prospectively acquired data obtained from two large North American cohorts. Both studies were IRB-approved at all clinical centers, elicited written informed consent from all participants, and were compliant with the Health Insurance Portability and Accountability Act (HIPAA).

COPDGene (ClinicalTrials.gov Identifier: NCT 00608764) is an ongoing NIH-sponsored, prospective, multicenter (*n* = 21), observational cohort study starting in November 2007 and consisting of more than 10,000 individuals who were current or former smokers at the time of enrollment. COPDGene aims to understand the etiology, progression, and heterogeneity of COPD (Regan et al., 2010). Inclusion criteria were age 45–80 years old at baseline visit, > 10 pack-years cigarette smoking history, and non-Hispanic white or African American race. Exclusion criteria were other lung diseases, pregnancy, cancer other than skin cancer in the 5 years prior to study entry, receiving antibiotics for a COPD exacerbation in the month prior to study enrollment, and relative of a previously enrolled participant.

SPIROMICS (ClinicalTrials.gov Identifier: NCT 01969344) is an ongoing NIH-sponsored prospective, multicenter (*n* = 12), observational cohort study starting in November 2010 and consisting of 2,981 current, former, and never-smokers at the time of enrollment (Couper et al., 2014). Inclusion criteria were age 40–80 years old at baseline visit, >20 pack-year cigarette smoking history for current or former smokers, and meeting lung function criteria based on spirometry without bronchodilators. An extensive list of exclusion criteria can be found on the ClinicalTrials.gov website (https://clinicaltrials.gov/ct2/show/NCT01969344).

Analysis utilized inspiratory and expiratory chest CT scans from both COPDGene (Regan et al., 2010; Han et al., 2011) and SPIROMICS cohorts (Sieren et al., 2016). In COPDGene, CT scanning occurred at three phases between 2007 and 2022. The intervals between phases were Phase 1–2 5.68 ± 0.89 (mean ± standard deviation, SD) years, Phase 2-3 4.60 ± 0.63 years, and Phase 1–3 10.09 ± 0.40 years. In SPIROMICS, CT scanning occurred at five timepoints between 2010 and 2022, with the baseline and fifth timepoint used in this analysis occurring 6.13 ± 1.05 years apart.

**FIGURE 1:**
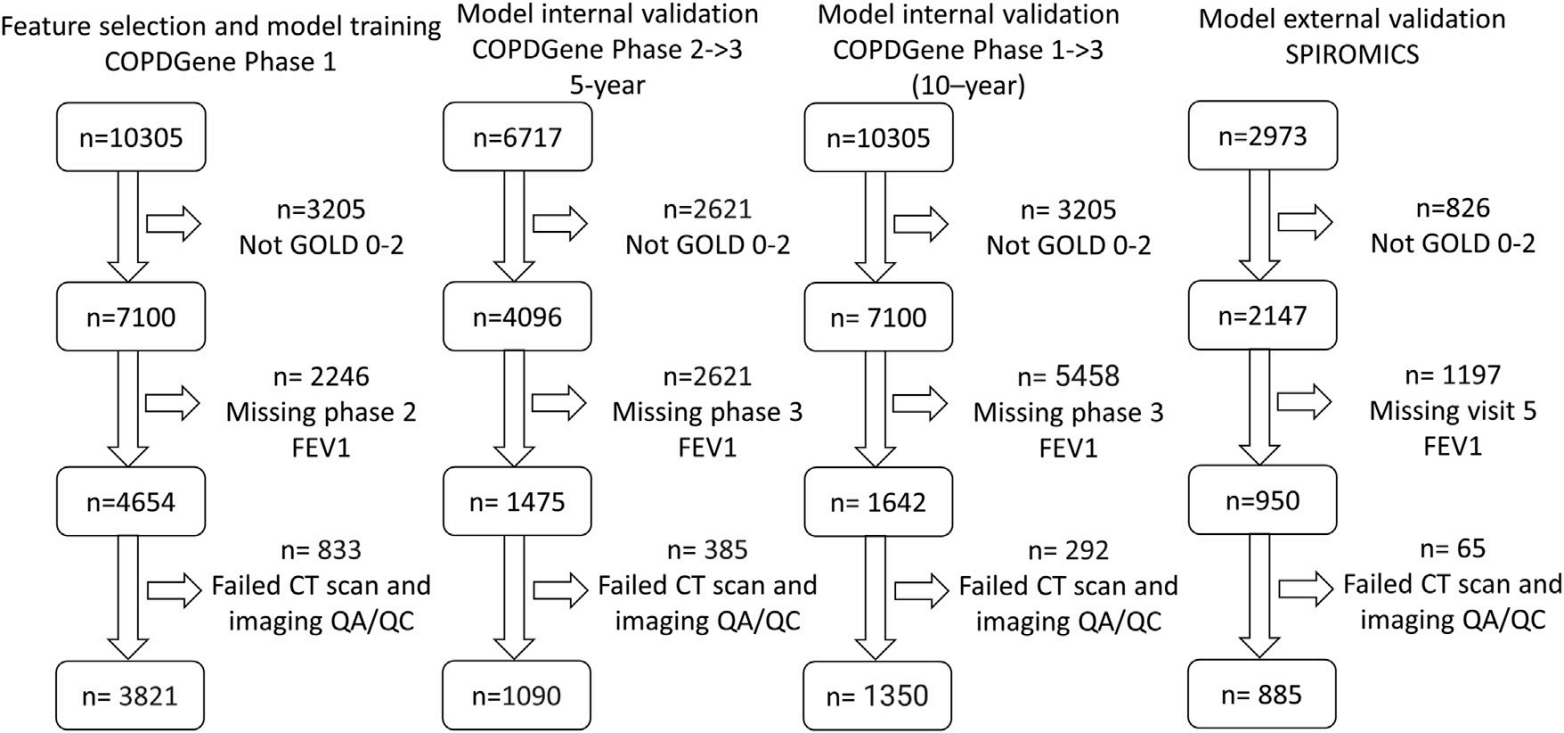
Consolidated Standards of Reporting Trials (CONSORT) diagram for model training. (COPDGene Phase 1), 5-year internal validation (COPDGene Phase 2–3), 10-year internal validation (COPDGene Phase 1–3), and external validation (SPIROMICS) datasets.

For both studies, COPD was defined by a post-bronchodilator FEV_1_/FVC (forced vital capacity) < 0.7 at the baseline visit, as specified in the GOLD guidelines at the time of study inclusion (Rabe et al., 2007). GOLD grades 1–4, based on post-bronchodilator spirometry, were used to define disease severity (GOLD 1, FEV_1_ ≥ 80% predicted; GOLD 2, FEV_1_ 50%–79% predicted; GOLD 3, FEV_1_ 30%–49% predicted; and GOLD 4, FEV_1_ < 30% predicted), with GOLD 0 classification defined by a post-bronchodilator FEV_1_/FVC ≥ 0.7 and FEV_1_% predicted ≥ 80%. We performed risk modeling based on GOLD category on the following numbers of participants: in COPDGene, GOLD 0 (*n* = 2,298) and GOLD 1–2 (*n* = 1,523), and in SPIROMICS, GOLD 0 (*n* = 385) and GOLD 1–2 (*n* = 500) (Figure 1; Tables 1, 2).

**TABLE 1:** Summary statistics for COPDGene Phase 1, COPDGene Phase 2, and SPIROMICS cohorts for GOLD 0 subjects.

	COPDGene P1	COPDGene P2	*p*-value COPDGene P2 vs. COPDGene P1	COPDGene P3	*p*-value COPDGene P3 vs. COPDGene P1	SPIROMICS	*p*-value SPIROMICS vs. COPDGene P1
GOLD 0 totals	2,298	665		839		385	
Rapid decline (>1.5% ∆ FEV_1_%pred)	26.5%	27.4%	*p = 0.62	12.8%	*p = 0.94	14.0%	*p = 0.71
Male	51.8%	55.0%	*p = 0.47	52.7%	*p = 0.58	55.8%	*p = 0.27
White	31.0%	25.6%	*p = 0.68	30.9%	*p = 0.68	49.3%	*p = 0.22
Current smokers	48.9%	33.5%	*p = 0.69	48.5%	*p = 0.61	42.9%	*p = 0.38
Lower lobe PRM_fSAD_ > 10%	10.6%	7.4	*p = 0.85	10.5%	*p = 0.81	6.2%	*p = 0.79
Upper lobe PRM_emphysema_ > 2%	14.4%	11.9	*p = 0.78	15.7%	*p = 0.75	10.1%	*p = 0.7
Age (years)	58.0 ± 8.5	**63.6 ± 8.3**	***p* < 0.001**	57.6 ± 8.3	*p* = 0.26	**61.9 ± 9.3**	***p* < 0.001**
Height (cm)	169.9 ± 9.4	**168.4 ± 9.2**	***p* < 0.001**	169.7 ± 9.2	*p* = 0.73	169.1 ± 9.4	*p* = 0.13
Weight (kg)	84.4 ± 18.4	83.4 ± 19.0	*p* = 0.25	83.8 ± 17.7	*p* = 0.39	84.0 ± 17.6	*p* = 0.68
FVC% predicted	97.5 ± 11.5	98.2 ± 11.7	*p* = 0.2	98.0 ± 11.5	*p* = 0.25	97.0 ± 12.4	*p* = 0.25
Post-bronchodilator FEV_1_/FVC	0.8 ± 0.1	0.8 ± 0.5	*p* = 0.16	0.8 ± 0.1	*p* = 0.58	**1.0 ± 0.1**	***p* < 0.001**
SGRQ score	14.8 ± 16.7	**12.8 ± 15.3**	***p* = 0.01**	14.2 ± 16.3	*p* = 0.36	**22.9 ± 17.1**	***p* < 0.001**
6MWD (m)	469.6 ± 106.5	**448.9+-120.7**	***p* < 0.001**	474.8 ± 110.2	*p* = 0.23	**438.3 ± 90.2**	***p* < 0.001**
Smoking pack-years	37.1 ± 20.3	38.1 ± 20.3	*p* = 0.25	36.5 ± 19.4	*p* = 0.51	**44.0 ± 27.8**	***p* < 0.001**
Inspiratory CT volume (mL)	5318.8 ± 1237.7	5273.9 ± 1261.7	*p* = 0.41	5325.2 ± 1251.6	*p* = 0.90	5226.4 ± 1253.5	*p* = 0.18
Expiratory CT volume (mL)	2639.3 ± 649.8	2600.3 ± 621.2	*p* = 0.17	2597.6 ± 652.4	*p* = 0.11	2685.6 ± 684.1	*p* = 0.2
TBV (mL)	172.9 ± 38.9	**169.3 ± 35.8**	***p* = 0.03**	175.7 ± 40.6	*p* = 0.08	171.0 ± 35.7	*p* = 0.35
tBV5 (%)	77.8 ± 4.0	**79.9 ± 4.1**	***p* < 0.001**	77.8 ± 4.1	*p* = 0.74	**79.4 ± 3.3**	***p* < 0.001**
trabecular.T12 (HU)	162.7 ± 50.5	**143.9 ± 41.8**	***p* < 0.001**	162.9 ± 48.7	*p* = 0.95	**151.4 ± 49.8**	***p* < 0.001**
cortical.T12 (HU)	332.7 ± 57.2	**316.4 ± 55.8**	***p* < 0.001**	333.6 ± 54.2	*p* = 0.69	**320.1 ± 54.8**	***p* < 0.001**

*The z-proportions test was used to compute *p*-values for binary variables. The unpaired *t*-test was used to compute *p*-values for continuous data. Bolded text indicates a *p*-value < 0.05. Abbreviations: SGRQ, St. George’s Respiratory Questionnaire; 6MWD, 6-min walk distance; TBV, total blood vessel volume; tBV5, ratio of vascular tree length in vessels with a cross-sectional area less than 5 mm^2^ to total vascular tree volume; trabecular.T12, average Hounsfield unit (HU) value within the trabecular region of the T12 vertebral body; cortical.T12, average Hounsfield unit (HU) value within the cortical region of the T12 vertebral body.

**TABLE 2:** Summary statistics for COPDGene Phase 1, COPDGene Phase 2, and SPIROMICS cohorts for GOLD 1–2 subjects.

	COPDGene P1	COPDGene P2	*p*-value COPDGene P2 vs. COPDGene P1	COPDGene P3	*p*-value COPDGene P3 vs. COPDGene P1	SPIROMICS	*p*-value SPIROMICS vs. COPDGene P1
GOLD 1–2 totals	1,523	425		511		500	
Rapid decline (>1.5% ∆ FEV_1_%pred)	29.8%	38.4%	*p = 0.49	25.2%	*p = 0.72	24.8%	*p = 0.72
Male	46.9%	43.8%	*p = 0.54	45.2%	*p = 0.6	41.6%	*p = 0.63
White	81.0%	80.9%	*p = 0.68	81.2%	*p = 0.73	70.0%	*p = 0.56
Current smokers	44.5%	34.4%	*p = 0.64	41.5%	*p = 0.62	36.6%	*p = 0.67
Lower lobe PRM_fSAD_ > 10%	59.8%	60.5%	*p = 0.45	55.6%	*p = 0.57	52.6%	*p = 0.59
Upper lobe PRM_emphysema_ > 2%	55.7%	55.8%	*p = 0.47	57.5%	*p = 0.53	52.8%	*p = 0.57
Age (years)	63.0 ± 8.5	**67.8 ± 8.1**	***p* < 0.001**	62.5 ± 8.1	*p* = 0.23	**65.6 ± 7.6**	***p* < 0.001**
Height (cm)	170.1 ± 9.7	170.3 ± 9.8	*p* = 0.68	170.6 ± 9.5	*p* = 0.31	170.8 ± 9.3	*p* = 0.15
Weight (kg)	82.3 ± 18.2	81.6 ± 18.3	*p* = 0.45	81.6 ± 17.8	*p* = 0.41	82.0 + 17.7	*p* = 0.70
FVC% predicted	72.8 ± 14.3	**74.4 ± 15.8**	***p* = 0.05**	**75.0 ± 15.4**	***p* < 0.001**	**75.2 ± 14.8**	***p* < 0.001**
Post-bronchodilator FEV_1_/FVC	0.6 ± 0.1	0.6 ± 0.1	*p* = 0.79	0.6 ± 0.08	*p* = 0.84	**0.8 ± 0.1**	***p* < 0.001**
SGRQ score	26.1 ± 20.7	**23.0 ± 19.0**	***p* = 0.01**	**23.4 ± 19.9**	***p* = 0.01**	**29.9 ± 16.5**	***p* < 0.001**
6MWD (m)	431.2 ± 109.4	424.1 ± 115.2	*p* = 0.24	**449.4 ± 105.5**	***p* < 0.001**	421.9 ± 98.3	*p* = 0.09
Smoking pack-years	49.1 ± 25.1	49.5 ± 23.7	*p* = 0.75	47.7 ± 23.0	*p* = 0.27	51.4 ± 25.3	*p* = 0.08
Inspiratory CT volume (mL)	5788.4 ± 1384.2	5878.2 ± 1438.3	*p* = 0.24	5890.9 ± 1432.1	*p* = 0.15	5896.5 ± 1418.4	*p* = 0.13
Expiratory CT volume (mL)	3312.9 ± 860.5	3311.7 ± 842.0	*p* = 0.98	3278.6 ± 856.8	*p* = 0.44	**3425.1 ± 886.1**	***p* = 0.01**
TBV (mL)	179.0 ± 39.5	176.3 ± 37.8	*p* = 0.21	180.4 ± 38.8	*p* = 0.50	182.6 ± 38.0	*p* = 0.08
tBV5 (%)	78.2 ± 4.2	**79.9 ± 3.9**	***p* < 0.001**	78.1 ± 4.3	*p* = 0.49	**78.0 ± 3.4**	***p* < 0.001**
trabecular.T12 (HU)	141.4 ± 71.0	**133.3 ± 44.6**	***p* = 0.03**	143.2 ± 47.6	*p* = 0.59	**133.6 ± 42.1**	***p* = 0.02**
cortical.T12 (HU)	319.5 ± 62.4	**312.5 ± 59.3**	***p* = 0.04**	320.3 ± 57.1	*p* = 0.8	**313.5 ± 59.8**	*p* = 0.06

*The z-proportions test was used to compute *p*-values for binary variables. The unpaired *t*-test was used to compute *p*-values for continuous data. Bolded text indicates a *p*-value < 0.05. Abbreviations: SGRQ, St. George’s Respiratory Questionnaire; 6MWD, 6-min walk distance; TBV, total blood vessel volume; tBV5, ratio of vascular tree length in vessels with a cross-sectional area less than 5 mm^2^ to total vascular tree volume; trabecular.T12, average Hounsfield unit (HU) value within the trabecular region of the T12 vertebral body; cortical.T12, average Hounsfield unit (HU) value within the cortical region of the T12 vertebral body.

### Image processing

For all scans, Imbio Inc. Lung Density Analysis version 3.1 (Minneapolis, MN, United States) was used to perform PRM, lung volume measurements, TBV, and tBV5 (the ratio of vascular tree length in vessels with a cross-sectional area less than 5 mm^2^ to total vascular tree volume). PRM_emphysema_ is defined as the percentage of lung voxels less than −950 Hounsfield units (HU) on the inspiratory CT scan and less than −856 HU on the expiratory CT scan following deformable registration; PRM_fSAD_ is defined as the percentage of lung voxels greater than or equal to −950 HU on the inspiratory CT scan but less than −856 HU on the expiratory CT. PRM metrics were binarized into clinically significant (PRM_emphysema_ > 2.0%, PRM_fSAD_ >10.0%) and non-clinically significant categories. The 2% and 10% thresholds were chosen by finding the thresholds that had the highest combination of sensitivity and specificity for univariate prediction of rapid decline in GOLD 0–2 subjects. Bone Mineral Density Analysis v.0.1 (Imbio Inc.) was used to compute the average HU value within the trabecular region of the T12 vertebral body (BMD_T12_). Airway analysis metrics such as mean segmental AWT and AWT for all airways with an internal perimeter of 10 mm (Pi10) were computed and analyzed in the feature selection step, but could not be used because different software vendors processed data for COPDGene (Thirona) and SPIROMICS (Vida Diagnostics), and thus, the high inter-software variability associated with airway measurements precluded comparisons of models using this data.

At the time this study was conducted, co-author CH, who performed the following statistical analysis and developed the machine learning models, was employed by Imbio Inc. Neither Imbio nor CH had influence over participant inclusion.

### Definition of accelerated FEV_1_ decline

There is no universally accepted definition of accelerated FEV_1_ decline. Martinez et al. defined it as 60 mL/year, roughly double the normal rate of decline in non-smokers (Martinez et al., 2018). However, in the COPDGene cohort, significantly more men than women fit this categorization. To develop sex-agnostic models, we instead categorized rapid progression as a drop of more than 1.5% FEV_1_ percent predicted per year ( ∆ FEV_1_%pred), which roughly corresponds to the top quartile of decline. Because a competing risk for accelerated decline is mortality, models were trained to classify participants at-risk-for rapid FEV_1_ decline given survival.

### Feature selection

**FIGURE 2:**
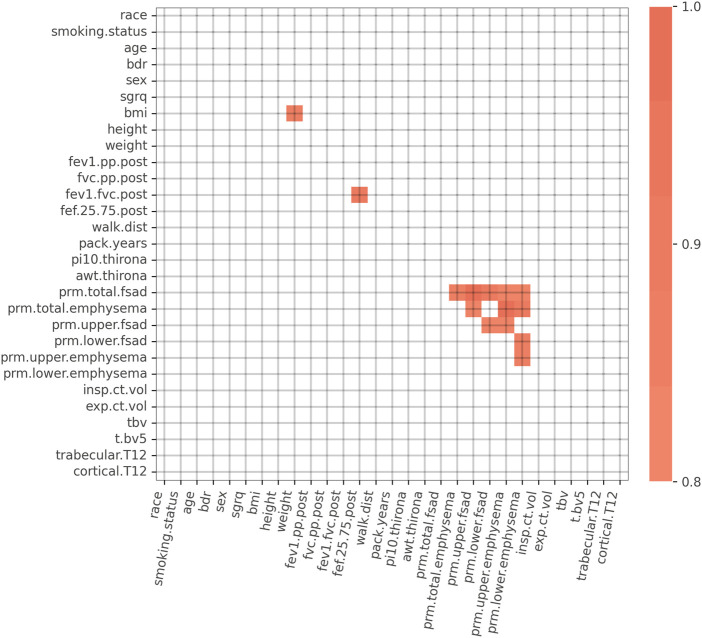
Spearman correlation matrix for the initial 29 variables. Spearman correlations > 0.80 are highlighted in orange. Strong correlations exist between PRM imaging biomarker variables, FEV_1_/FVC and FEF_25-75_, and BMI and weight. Abbreviations: bdr, bronchodilator responsiveness; sgrq, St. George’s Respiratory Questionnaire; BMI, body mass index; FEV1.pp.post, post bronchodilator FEV_1_% predicted; FVC.pp.post, post bronchodilator FVC % predicted; FEV1.FVC.post, post bronchodilator FEV_1_/FVC; FEF.25.75.post, post bronchodilator forced mid-expiratory flow; walk.dist, 6-min walk distance; pi10.thirona, square root of an airway wall area with a 10 mm lumen perimeter, measured by Thirona software; awt.thirona, segmental airway wall thickness, measured by Thirona software; prm.total.fsad, PRM total fSAD > 10%; prm.total.emphysema, PRM total emphysema > 2%; prm.upper.fsad, PRM upper lobes fSAD > 10%; prm.lower.fsad, PRM lower lobes fSAD > 10%; prm.upper.emphysema, PRM upper lobes emphysema > 2%; prm.lower.emphysema, PRM lower lobes emphysema > 2%; insp.ct.vol, CT-derived inspiratory lung volume; exp.ct.vol, CT-derived expiratory lung volume; tbv, total blood vessel volume; t.bv5, ratio of vascular tree length in vessels with a cross-sectional area less than 5 mm^2^ to total vascular tree volume; trabecular.T12, average Hounsfield unit (HU) value within the trabecular region of the T12 vertebral body; cortical.T12, average Hounsfield unit (HU) value within the cortical region of the T12 vertebral body.

We initially considered a set of K = 29 features consisting of symptoms, spirometry, demographic, and CT imaging biomarker data (Figure 2). To simplify the models and to increase reliability of the inputs, we chose to not use self-reported data. A 29 × 29 table of Spearman correlations between each feature was generated to assess feature redundancy (Parr et al., 2020) using data from GOLD 0–2 subjects. Features that had a Spearman correlation > 0.80 with any other variables were considered for removal due to information redundancy. For example, we retained weight and height but removed body mass index (BMI) because weight and height were independent from each other, but weight was highly correlated to BMI. Forced mid-expiratory flow (FEF_25.75_) was removed because it correlated strongly with FEV_1_/FVC. Finally, of the PRM imaging biomarker variables, PRM_upper lobes emphysema_ and PRM_lower lobes fSAD_ had the lowest correlation and thus were retained.

Following manual feature pruning, we conducted a “drop-column” feature-importance ranking and data-driven pruning procedure for the remaining 22 parameters. Classification models were trained using COPDGene Phase 1 data, with the outcome ∆ FEV_1_%pred variable measured between Phase 1 and Phase 2. We generated an initial model using all K = 22 features to arrive at a baseline model performance based on the receiver operating characteristic area under the curve (ROC-AUC). To arrive at AUC distribution, model performance was assessed using repeat cross-validation (M = 5,000 cross-validations with a train/test ratio of 80%/20%). The same procedure was repeated K = 22 times, each time removing a single feature, which produced a distribution of AUC reductions for each instance of feature removal. Features that resulted in a decrease in model AUC performance when “dropped” were retained for use in the final model; all others were removed (Figure 2). The drop-column procedure was performed separately for GOLD 0 and GOLD 1–2 subjects, resulting in two distinct variables sets for the final models.

In addition to a full variable model considering all variables that survived feature selection, we also created limited models using only imaging biomarkers (i.e., lung volumes, PRM, vascular, and bone density measurements) and data readily available from the DICOM header (i.e., age, sex), referred to hereafter as “CT-limited model.” The purpose of this analysis was to determine the predictive value of data that could only be obtained from a CT scan file within the electronic medical record.

### Machine learning methods and parameters

A wide variety of machine-learning techniques can be used to predict biological outcomes from multiple sources of data (Garavand et al., 2022). In this study, models were developed and validated using logistic regression with an L1 regularization penalty (a.k.a. LASSO) due to simplicity of implementation (i.e., few hyper-parameters) compared to other popular machine learning frameworks such as Random Forests or XGBoost. The L1 regularization penalty was employed to learn models that have a sparse set of significant predictor variables. We used the scikit-learn software package v1.0.2 within Python version 3.8.10 for all model development and validation. Model hyper-parameters were optimized using a Randomized Cross Validation search algorithm (sklearn.model_selection.RandomizedSearchCV). The cross validation hyper-parameter search resulted in an optimal inverse regularization penalty weight of C = 0.3.

### Internal and external validation

Internal validation was performed in two ways. First, we applied the models developed on the COPDGene Phase 1 training data to the COPDGene Phase 2 data, where the output variable was the ∆ FEV_1_%pred between the Phase 2 and Phase 3 visits. Second, we validated the model developed on the COPDGene Phase 1 data against the ∆ FEV_1_%pred between the Phase 1 and Phase 3 data, which were roughly 10 years apart. External validation was performed on data from the SPIROMICS study. Differences in the distribution between data in the training, internal validation, and external validation cohorts are shown in Table 1. Confidence intervals were computed by computing 1.96 × SD of the AUC using bootstrapping with 5,000 resamples. Permutation testing was used to generate AUC *p*-values.

### Relative risk based on model output probabilities

Following model generation, we recorded the logistic regression output probabilities associated with the lower 25th (p_25_) and upper 75th (p_75_) percentiles of the training data. We computed the relative risk between participants with FEV_1_%pred decline risk > p_75_ and those with risk < p_25_ for the training dataset (COPDGene Phase 1-Phase 2), the internal validation dataset (COPDGene Phase 2-Phase 3), and the external validation dataset. Additionally, we looked at the relative risk of rapid decline over 10 years by examining subjects that had ∆ FEV_1_%pred data between COPDGene Phase 1 and Phase 3. Relative risk was computed using the scipy.stats.contingency.relative_risk software package v1.8.1.

## Results

Results are separated into 3 broad sections: 1) feature selection to create the training model, 2) within the COPDGene cohort cross validation and 3) external SPIROMICS cohort validation, followed by a section on relative risk.

### Within cohort analysis

**FIGURE 3:**
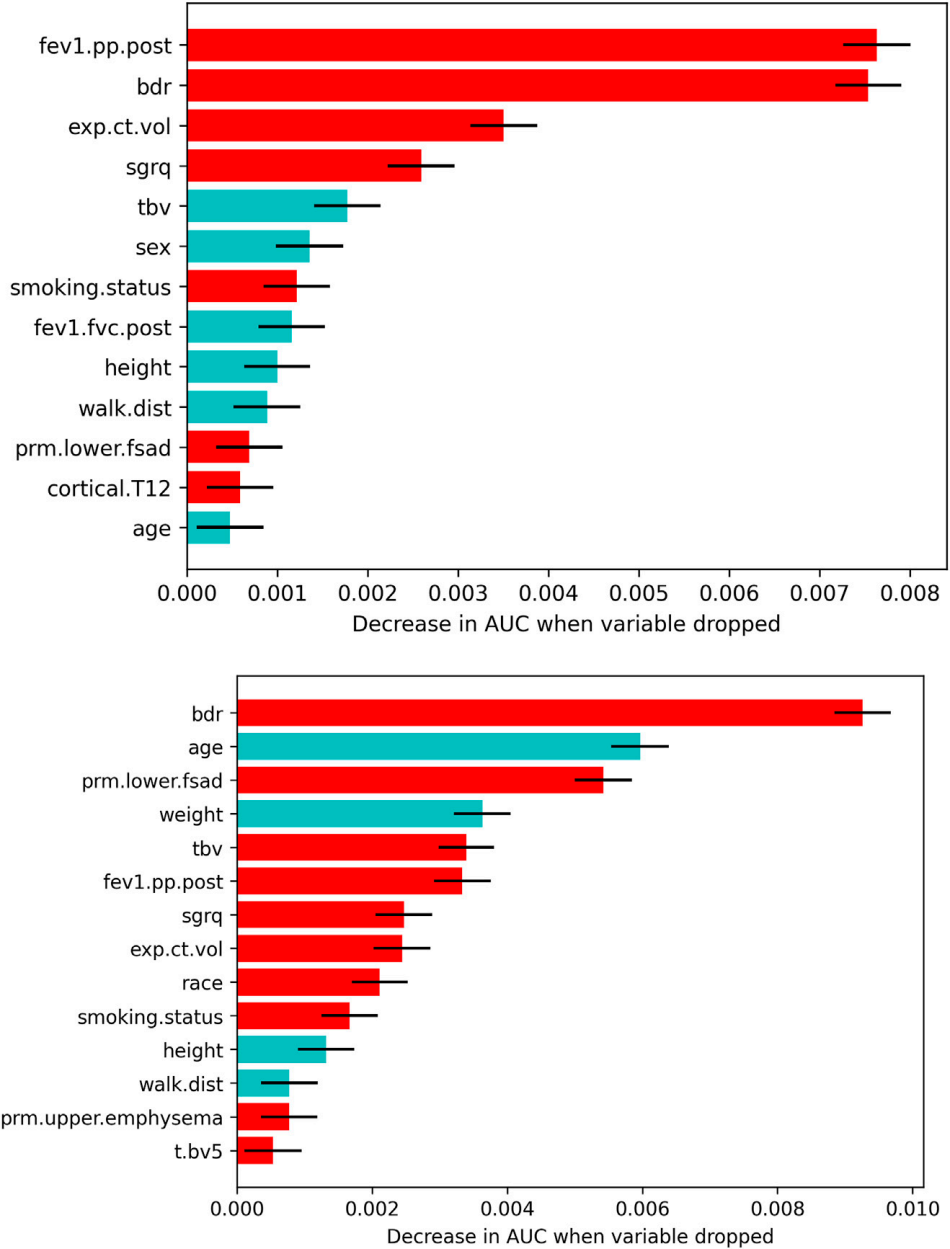
Features that survived the drop-column variable selection procedure for each GOLD group and model type (top: GOLD 0; bottom: GOLD 1–2). The mean ±1.96 × SD decrease in AUC related to dropping each feature is plotted only for features that significantly affected the model. Red bars indicate a higher risk of rapid FEV_1_ decline with increasing value, having bronchodilator reversibility, African American race, and/or being a current smoker. Blue bars indicate a higher risk of rapid FEV_1_ decline with decreasing value and male sex. Abbreviations: fev1.pp.post, post bronchodilator FEV_1_% predicted; bdr, bronchodilator responsiveness; exp.ct.vol, CT-derived expiratory lung volume; sgrq, St. George’s Respiratory Questionnaire; tbv, total blood vessel volume; fev1.fvc.post, post bronchodilator FEV_1_/FVC; walk.dist, 6-min walk distance; prm.lower.fsad, PRM lower lobes fSAD > 10%; cortical.T12, average HU value in the cortical region of the T12 vertebra; prm.upper.emphysema, PRM upper lobes emphysema > 2%; t.bv5, ratio of vascular tree length in vessels with a cross-sectional area less than 5 mm^2^ to total vascular tree volume.

Figure 3 depicts all features that survived the variable selection procedure during model training in models developed on COPDGene Phase 1-Phase 2 data. In the full-variable model for the GOLD 0 cohort, higher post bronchodilator FEV_1_% predicted (FEV_1_pp.post), bronchodilator responsiveness (BDR), greater expiratory CT volume, and a higher St. George’s Respiratory Questionnaire (SGRQ) score were the most important variables for predicting rapid FEV_1_ decline. Less significant variables included a lower TBV, male sex, and current smoking status.

In the full-variable model for the GOLD 1–2 cohort, BDR, younger age, PRM_lower lobes fSAD_ > 10%, and higher blood vessel volume were the most important variables predicting rapid FEV_1_ decline. Less significant variables included higher SGRQ score, lower weight, higher FEV_1_pp.post, current smoking status, higher expiratory CT volume, shorter height and 6-min walk distance (6MWD), African American race, and PRMupper lobes emphysema > 2%.

### Within cohort validation

Following model training, we validated the CT-limited COPDGene models using the same training variables within the COPDGene cohort. This process used data from both the 5-year (P2–P3) and 10-year (P1–P3) follow-up periods. For the GOLD 0 models, AUCs were similar to those from the training dataset, whereas the GOLD 1–2 models were still significant but had much lower AUCs during the 5-year follow up (P2–P3) than during training (Table 3).

**TABLE 3:** Cross-validation AUCs, listed as mean ± 1.96 standard deviation (SD).

	Full model*	CT-limited model**
5-year COPDGene P1–P2 (training)		
GOLD 0	0.653 ± 0.025 (*p* < 0.001)	0.592 ± 0.027 (*p* < 0.001)
GOLD 1–2	0.713 ± 0.028 (*p* < 0.001)	0.650 ± 0.030 (*p* < 0.001)
5-year COPDGene P2–P3 (Internal validation)		
GOLD 0	0.622 ± 0.048 (*p* < 0.001)	0.562 ± 0.048 (*p* = 0.028)
GOLD 1–2	0.644 ± 0.054 (*p* < 0.001)	0.591 ± 0.059 (*p* = 0.008)
10-year COPDGene P1–P3 (Internal validation)		
GOLD 0	0.680 ± 0.053 (*p* < 0.001)	0.603 ± 0.057 (*p* = 0.005)
GOLD 1–2	0.753 ± 0.047 (*p* < 0.001)	0.679 ± 0.028 (*p* < 0.001)
SPIROMICS (external validation)		
GOLD 0	0.622 ± 0.081 (*p* = 0.021)	0.571 ± 0.089 (*p* = 0.120)
GOLD 1–2	0.640 ± 0.059 (*p* < 0.001)	0.568 ± 0.059 (*p* = 0.056)

*GOLD 0 full model variables: post bronchodilator FEV_1_% predicted, BDR, CT-derived expiratory lung volume, SGRQ score, TBV, sex, smoking status, post bronchodilator FEV1/FVC, height, 6MWD, PRMlower lobes fSAD > 10%, cortical.T12, age.

*GOLD 1–2 full model variables: BDR, age, PRM_lower lobes fSAD_ > 10%, weight, TBV, post bronchodilator FEV_1_% predicted, SGRQ score, CT-derived expiratory lung volume, race, smoking status, height, 6MWD, PRM_upper lobes emphysema_ > 2%, tBV5.

**GOLD 0 CT-limited model variables: CT-derived expiratory lung volume, TBV, sex, PRM_lower lobes fSAD_ > 10%, cortical.T12, age.

**GOLD 1–2 CT-limited model variables: age, PRM_lower lobes fSAD_ > 10%, TBV, CT-derived expiratory lung volume, PRM_upper lobes emphysema_ > 2%, tBV5.

Surprisingly, AUCs increased for all models in the 10-year validation (P1–P3). This result is atypical in machine learning, as model AUCs tend to be highest in training data. A possible explanation is that we employed the same pool of independent variables used for training to predict the same signal, but over a longer period of time. This process might decrease the noise inherent in serial FEV_1_ measurements, and thus increase the prediction accuracy.

### External cohort validation

External validation on SPIROMICS data resulted in trends similar to COPDGene P2–P3 data, showing slight predictive drops in performance. The full variable models were significant (*p* < 0.05) for both the GOLD 0 and GOLD 1–2 groups; however, the performance of the CT-limited models declined and were no longer significant for GOLD 0 participants. A possible explanation is that there were only roughly half as many SPIROMICS participants as in the COPDGene P2–P3 analysis.

In general, GOLD 1–2 models consistently performed better than GOLD 0 models. This finding suggests that accelerated lung function decline can be predicted with greater accuracy in those who already have spirometric evidence of airflow obstruction. Additionally, the prediction accuracy of the CT-limited models was much lower than the full variable models across all training and validation groups.

### Relative risk

We next computed relative risk between two groups: those with rapid decline risk greater than the upper 75th (p_75_) versus those with decline less than the lower 25th (p_25_) (Table 4). For this purpose, we used the logistic regression output probabilities of the training data for all four datasets. The highest relative risk was consistently seen in the 10-year internal validation group (COPDGene P1–P3), suggesting that over longer follow-up, these predictive models can effectively discriminate those at highest risk of accelerated lung function decline.

**TABLE 4:** Relative risk of rapid FEV_1_ decline associated with risk score greater than the upper 75th percentile (p_75_) versus risk less than the lower 25th (p_25_).

	Full model*		CT-limited model**	
	GOLD 0	GOLD 1, 2	GOLD 0	GOLD 1, 2
Training data COPDGene P1–P2	2.6 (2.1, 3.3)	3.7 (2.8, 4.9)	1.9 (1.5, 2.3)	2.7 (2.1, 3.5)
Internal validation COPDGene P2–P3 (5 year)	2.0 (1.4, 2.9)	2.5 (1.8, 3.5)	1.5 (1.1, 2.2)	1.3 (1.0, 1.6)
Internal validation COPDGene P1–P3 (10-year)	5.8 (2.8, 12.0)	8.1 (3.8, 16.9)	2.4 (1.5, 3.6)	3.5 (2.0, 5.6)
External validation SPIROMICS	2.5 (0.99, 6.3)	1.8 (1.16, 2.8)	2.14 (1.1, 4.1)	1.6 (1.0, 2.4)

95% confidence intervals are in parentheses.

*GOLD 0 full model variables: post bronchodilator FEV_1_% predicted, BDR, CT-derived expiratory lung volume, SGRQ score, TBV, sex, smoking status, post bronchodilator FEV_1_/FVC, height, 6MWD, PRM_lower lobes fSAD_ > 10%, cortical.T12, age.

*GOLD 1–2 full model variables: BDR, age, PRM_lower lobes fSAD_ > 10%, weight, TBV, post bronchodilator FEV_1_% predicted, SGRQ score, CT-derived expiratory lung volume, race, smoking status, height, 6MWD, PRM_upper lobes emphysema_ > 2%, tBV5.

**GOLD 0 CT-limited model variables: CT-derived expiratory lung volume, TBV, sex, PRM_lower lobes fSAD_ > 10%, cortical.T12, age.

**GOLD 1–2 CT-limited model variables: age, PRM_lower lobes fSAD_ > 10%, TBV, CT-derived expiratory lung volume, PRM_upper lobes emphysema_ > 2%, tBV5.

Focusing only on GOLD 1–2 participants in the COPDGene P1–P3 group, the full model can predict an over 8-fold increased risk of accelerated FEV_1_ decline among p_75_ versus p_25_ participants. Relative risk was lower in all the tested cohorts when using the CT-limited model, consistent with prior results in this analysis.

Overall, these relative risk profiles suggest that the full variable models could effectively identify those at highest risk of lung function decline with sufficient accuracy to provide clinical relevancy and utility in decision making. By contrast, the CT-limited models lack significantly increased relative risk in the SPIROMICS data, despite promising results in the COPDGene 10-year analysis. This finding highlights the need for further external validation using 10-year follow-up data to determine more conclusively whether screening for rapid FEV_1_ decline will be practically feasible using only data from DICOM files.

## Discussion

We studied the important clinical question of whether machine learning techniques can predict which ever-smokers, either at-risk-for COPD or with mild-moderate disease, are at highest risk for accelerated FEV_1_ decline. In an ideal world, one may imagine that identifying individuals at-risk-for disease progression only from data available in a chest CT would allow for increased availability of data to clinicians in a potentially automated fashion. Using logistic regression models built based on a combination of radiographic and clinical features, we show that this approach is feasible.

We were able to identify the most relevant imaging biomarker features predicting accelerated lung function decline, which importantly differed between those with established disease and those at-risk. Key variables in GOLD 1–2 participants included PRM_lower lobes fSAD_ and demographic information such as age, expiratory lung volume in GOLD 0 participants, and pulmonary function results, particularly BDR, in both groups. Our models showed only modest decreases in predictive strength, as indicated by AUC analysis, in the external validation cohort, and improved at longer intervals in the training set (COPDGene), for which longer follow-up data were available. In the latter group, the full variable models were able to predict an 8-fold difference in relative risk between those in the highest versus lowest quartile of lung function decline. These findings support the use of combined radiographic and clinical data models to select participants for therapeutic trials of potentially disease-modifying agents in COPD.

A strength of this analysis is that the input variables in the full models are objective, and most are readily available, due to the use of spirometry rather than more complex pulmonary function testing and to the fact that many individuals also have recent CT scans. In the CT-limited model, additional variables, including TBV, were also found to be significant contributors to prediction of accelerated FEV_1_ decline, though with interesting differences based on COPD status. Thus, FEV_1_ decline was associated with lower TBV in GOLD 0, but with higher TBV in GOLD 1–2. One possible explanation for this disparity is the known association of distal pruning of pulmonary vessels with early COPD progression (Estepar et al., 2013; Synn et al., 2019; Weatherald et al., 2019). However, further studies are needed to understand why this relationship is reversed in later disease stages, where there may be additional anatomic changes associated with development of pulmonary hypertension (Elwing and Panos, 2008).

Our finding of the strong predictive impact of BDR is also noteworthy, as BDR has recently been associated with thicker airway walls in COPD subjects and decline in lung function (Donaldson et al., 2005; Kim et al., 2014). This may be due to active inflammatory disease at the level of the distal, smaller airways, leading to loss of lung function over time (Barjaktarevic et al., 2019).

Our results agree with a recent study that used similar machine learning techniques to predict progression of FEV_1_ in COPDGene (Boueiz et al., 2022) and that also utilized P1–P2 and P1–P3 data for internal validation. Their machine learning models, both logistic regression and random forest, were able to predict an absolute cross-sectional FEV_1_ at follow-up visits with excellent results (ROC-AUCs > 0.9). However, similar to our work, they had greater difficulty predicting change in FEV_1_ over time, with ROC-AUCs around 0.7 for both types of their models. This may be due to the fact that the relative changes in FEV_1_ over a few years are small and result in low signal-to-noise ratios. We extend those findings via use of novel PRM and vascular biomarkers in our prediction models, as well as by external validation in a separate cohort to improve their performance despite these inherent limitations.

The overall modest AUCs across our models, ranging roughly between 0.5–0.7, imply that FEV_1_ is an imprecise (i.e., noisy) dependent variable. Models trained on noisy binary classification outcomes can still converge upon an optimal solution, as long as noise is symmetric, i.e., the same odds of false positive and false negative measurements, and there are sufficient observations (Lugosi, 1992). That AUCs were highest for GOLD 1-2 participants was expected, and in agreement with the analysis of Boueiz et al. (2022), as FEV_1_ decline in COPD is most rapid in this group, making the signal-to-noise ratio highest (D'Amato et al., 2016). Also as expected, we found that pulmonary function, symptoms, and more extensive demographic data improved prediction accuracy over using imaging variables alone. In particular, BDR was consistently the most important non-imaging variable, with SGRQ score also providing significant information.

That some of our findings were no longer statistically significant in the CT-limited model for GOLD 0 indicates that screening this group using imaging data alone is inadequate. These findings further emphasize the challenge of predicting FEV_1_ decline in ever-smokers without airflow obstruction. While currently available clinical parameters alone do not adequately predict risk in this group, it is promising that when combined with radiographic variables, these novel machine learning techniques can be harnessed to assist in clinical decision making in a relatively short period of time. There remains great potential to harness these techniques for a variety of clinical applications to fill patient care gaps. For example, the ability to better phenotype patients will allow researchers to identify suitable subjects for clinical trials, target subjects at greatest risk for accelerated lung function decline, and more rapidly assess response to new therapies.

We also acknowledge several limitations to our work and have identified key goals moving forward. We only tested logistic regression models using machine learning techniques for feature selection. Although we did not test other algorithms, notably decision tree models, we replicated findings of a recent study that did. Prior studies have attempted to phenotype subjects at risk of lung function decline using machine learning methods such as decision tree models in other cohorts, but did not adjust for decline associated with sex, which was the greatest predictor of decline (Nikolaou et al., 2021). As stated above, prediction of FEV_1_ remains challenging, and continued refinement and optimization of our models will be required before these techniques can be applied clinically to account for lung function decline that may be fairly subtle over a few years. A wealth of biomarker data correlates to risk of COPD progression, exacerbations, and mortality (Leitao Filho et al., 2020; Tanimura et al., 2020; Pratte et al., 2021; Serban et al., 2021; Singh et al., 2021) that was not included in our current models, but in future iterations would be important to consider. Survivorship bias is another limitation in longitudinal observational cohort studies, especially in subjects with advanced COPD and increased short term mortality risk, that may influence our results. Finally, most of the longitudinal follow-up in this study occurred over a 5-year period, with some additional analysis based on 10-year follow-up data. However, a longer follow-up period may further enhance our model’s performance and predictive ability.

In summary, we determined that a combination of radiographic and clinical variables can help predict which individuals are at highest risk for rapid FEV_1_ decline. While logistic regression models trained on a limited but more easily obtainable dataset captured from CT scans also had significant predictive value, these models were not as accurate. Predicting FEV_1_ decline continues to be challenging and there remains a strong unmet clinical need for further refinement of these models.

## Figures and Tables

**Table:** 

**6MWD**	6-min walk distance
**AUC**	area under the curve
**AWT**	airway wall thickness
**awt.Thirona**	segmental airway wall thickness, measured by Thirona software
**BDR**	bronchodilator responsiveness
**BMI**	body mass index
**CONSORT**	Consolidated Standards of Reporting Trials
**COPD**	chronic obstructive pulmonary disease
**COPDGene**	Genetic Epidemiology of Chronic Obstructive Pulmonary Disease
**cortical.T12**	average Hounsfield unit value within the cortical region of the T12 vertebral body
**CT**	computed tomography
**DICOM**	Digital Imaging and Communications in Medicine
**exp.ct.vol**	CT-derived expiratory lung volume
**FEF**_**25.75**_	forced mid-expiratory flow
**FEF.25.75.post**	post bronchodilator forced mid-expiratory flow
**FEV**_**1**_	forced expiratory volume in 1 s
**FEV**_**1**_ **.FVC.post**	post bronchodilator FEV_1_/FVC
**FEV**_**1**_ **.pp.post**	post bronchodilator FEV_1_% predicted
∆ **FEV**_**1**_ **%pred**	FEV_1_ percent predicted per year
**fSAD**	functional small airways disease
**FVC**	forced vital capacity
**FVC.pp.post**	post bronchodilator FVC % predicted
**GOLD**	Global Initiative for Chronic Obstructive Lung Disease
**HIPAA**	Health Insurance Portability and Accountability Act
**HU**	Hounsfield unit
**insp.ct.vol**	CT-derived inspiratory lung volume
**IRB**	institutional review board
**PFT**	pulmonary function testing
**Pi10**	square root of an airway wall area with a 10 mm lumen perimeter
**Pi10.thirona**	square root of an airway wall area with a 10 mm lumen perimeter, measured by Thirona software
**PRM**	Parametric Response Mapping
**prm.lower.emphysema**	PRM lower lobes emphysema > 2%
**prm.lower.fsad**	PRM lower lobes fSAD > 10%
**prm.total.emphysema**	PRM total emphysema > 2%
**prm.total.fsad**	PRM total fSAD >10%
**prm.upper.emphysema**	PRM upper lobes emphysema > 2%
**prm.upper.fsad**	PRM upper lobes fSAD > 10%
**ROC-AUC**	receiver operating characteristic area under the curve
**SD**	standard deviation
**SGRQ**	St. George’s Respiratory Questionnaire
**SPIROMICS**	Subpopulations and Intermediate Outcome Measures in COPD
**TBV**	total blood vessel volume
**tBV5/t.bv5**	ratio of vascular tree length in vessels with a cross-sectional area less than 5 mm^2^ to total vascular tree volume
**trabecular.T12**	average Hounsfield unit value within the trabecular region of the T12 vertebral body
**walk.dist**	6-min walk distance

## Data Availability

The data analyzed in this study is subject to the following licenses/restrictions: The datasets presented in this study are not readily available because they are part of NIH sponsored clinical trials and require a data-use agreement to be signed. For access to COPDGene data visit https://www.copdgene.org/phase-1-study-documents.htm for instructions. For access to SPIROMICS data, visit https://www.spiromics.org/spiromics/obtaining-data for instructions. Requests to access these datasets should be directed to COPDGene: https://www.copdgene.org/phase-1-study-documents.htm; SPIROMICS: https://www.spiromics.org/spiromics/obtaining-data.
